# Predicting the Physiological Role of Circadian Metabolic Regulation in the Green Alga *Chlamydomonas reinhardtii*

**DOI:** 10.1371/journal.pone.0023026

**Published:** 2011-08-22

**Authors:** Sascha Schäuble, Ines Heiland, Olga Voytsekh, Maria Mittag, Stefan Schuster

**Affiliations:** 1 Department of Bioinformatics, Friedrich Schiller University Jena, Jena, Germany; 2 Institute of General Botany and Plant Physiology, Friedrich Schiller University Jena, Jena, Germany; Instituto de Biología Molecular y Celular de Plantas, Spain

## Abstract

Although the number of reconstructed metabolic networks is steadily growing, experimental data integration into these networks is still challenging. Based on elementary flux mode analysis, we combine sequence information with metabolic pathway analysis and include, as a novel aspect, circadian regulation. While minimizing the need of assumptions, we are able to predict changes in the metabolic state and can hypothesise on the physiological role of circadian control in nitrogen metabolism of the green alga *Chlamydomonas reinhardtii*.

## Introduction

Metabolic pathway analysis is a well established and very useful tool in Systems Biology [1], [2]. One concept in this field is that of elementary flux modes (EFMs), which represents a minimal set of reactions that can operate at steady state with all reactions proceeding in the thermodynamically feasible direction [3]. The EFM approach has proved its value in diverse biotechnological applications [4]. It has been used to find efficient routes for the production of particular target compounds, such as fatty acids in plants [5], or methionine [6] and cyanophycin [7] in bacteria, to find possible targets for the engineering of metabolic networks through knock-outs or knock-ins [8], [9], as well as to assess the impact of enzyme deficiencies [10], [11] or the robustness of metabolic networks [12]. Note that in contrast to optimality based approaches like Flux Balance Analysis [13], EFM analysis has the advantage of providing a more comprehensive overview of the existing routes through a given network by providing a complete data set of possible fluxes rather than solely an optimality restricted set. A disadvantage arises from the problem of combinatorial explosion [14]. Therefore, it is impossible to compute all EFMs in genome-scale models up to now, although advances have been made recently coping with large networks [15].

Beside a growing number of methods for the analysis of metabolic networks, connecting experimental data to reconstructed models remains a major task to systems biology [16]–[20]. However, this potential should not be underestimated, as immense data are produced by modern techniques, such as high throughput sequencing, as well as microarrays and proteomics. Moreover, inherent information in DNA sequences, like recognition motifs, can be utilised as well and ultimately applied to network analysis, linking genomics, proteomics and metabolomics. This offers an access to regulation processes that possibly lead to altered metabolic fluxes and consequently influence the entire metabolism of an organism.

To demonstrate the usefulness of our method with a case study, we describe the analysis of a reconstructed metabolic network of nitrogen uptake in the green algae *Chlamydomonas reinhardtii*, a model process for green crop plants. Assimilating nitrogen is a key step of metabolism required by phototrophic organisms in order to grow and survive in natural habitats [21]. Nitrogen metabolism in this green algae is circadian-clock regulated, via an mRNA binding factor named CHLAMY1, a heteromer that consists of two subunits, C1 and C3, the latter being well conserved in humans [22]. This regulator is known to bind UG-repeats that comprise at least seven non-interrupted UG-repetitions and are located in the 3′ UTR of various mRNAs including nitrite reductase and argininosuccinate lyase [22]–[24]. It has been shown experimentally that introduction of UG-repeats into the 3′ UTR of reporter constructs results in circadian expression [25]. The binding activity is controlled by the circadian clock, as it increases at the end of the day and decreases again at the end of the night. As activity levels of nitrite reductase, whose mRNA bears a UG-repeat, and of reporters that are under control of the UG-repeats are highest at the beginning of the day, it is assumed that CHLAMY1 binding prevents translation during the night [25], [26].

Here, we combine genome based sequence and metabolic pathway analyses by computing EFMs. This allows us to evaluate the changes in nitrogen assimilation and amino acid anabolism that are caused by CHLAMY1 binding and thus, determine the physiological role of this circadian RNA-binding factor. We study amino acid biosynthesis of alanine, glycine, asparagine, lysine and arginine, which permits physiological interpretation and comparison to known data from other organisms. These amino acids were chosen as they are either overrepresented in *C. reinhardtii* or contain a high nitrogen content in their side chain and, thus, are particularly suitable for nitrogen storage.

As we will show, the application of optimality principles that solely focus on analysing maximum yields like in Flux Balance Analysis [13], only offers a limited view on a given system and is therefore not suitable for our approach as the complete capability of the network has to be taken into account.

## Results

As it is not feasible to analyse the complete metabolism of *C. reinhardtii* using elementary flux mode analysis, we first had to confine our model. *C. reinhardtii* is able to grow either autotrophically, heterotrophically or mixotrophically. As we simulate only metabolism during the night here, we have chosen acetate and glucose-6-phosphate (G6P) as carbon sources. G6P is provided by starch degradation. The degradation is not explicitly included into the model.

**Figure 1:**
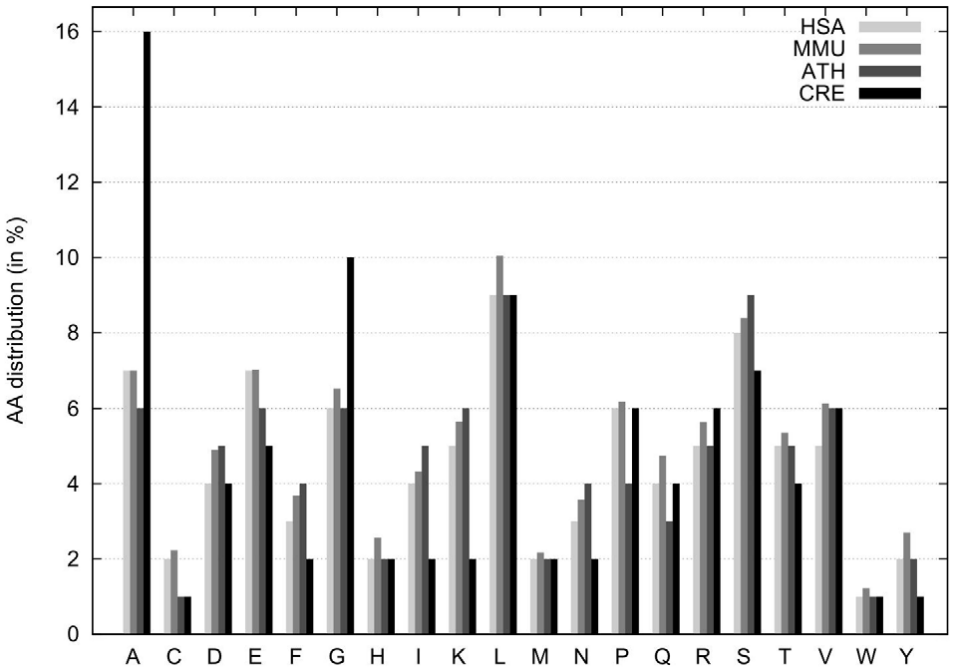
Distribution of amino acids among different species. Percentage share of amino acids are given for each amino acid using one letter code. The amino acid compositions of selected organisms were derived from complete genome ORF prediction from different databases (see Analysis section). HSA: *Homo sapiens*, MMU: *Mus musculus*, ATH: *Arabidopsis thaliana*, CRE: *Chlamydomonas reinhardtii*.

To model the nitrogen uptake, we analysed the biosynthesis of five different amino acids. First, we selected amino acids that have the highest nitrogen to carbon ratio, those are lysine, asparagine and arginine. Furthermore, we analysed the amino acid composition of all predicted proteins in *C. reinhardtii* and identified glycine and alanine as most abundant and highly overrepresented amino acids compared to other organisms (Fig. 1). Additionally, glutamate, glutamine and aspartate are present in the model as intermediates.

**Figure 2:**
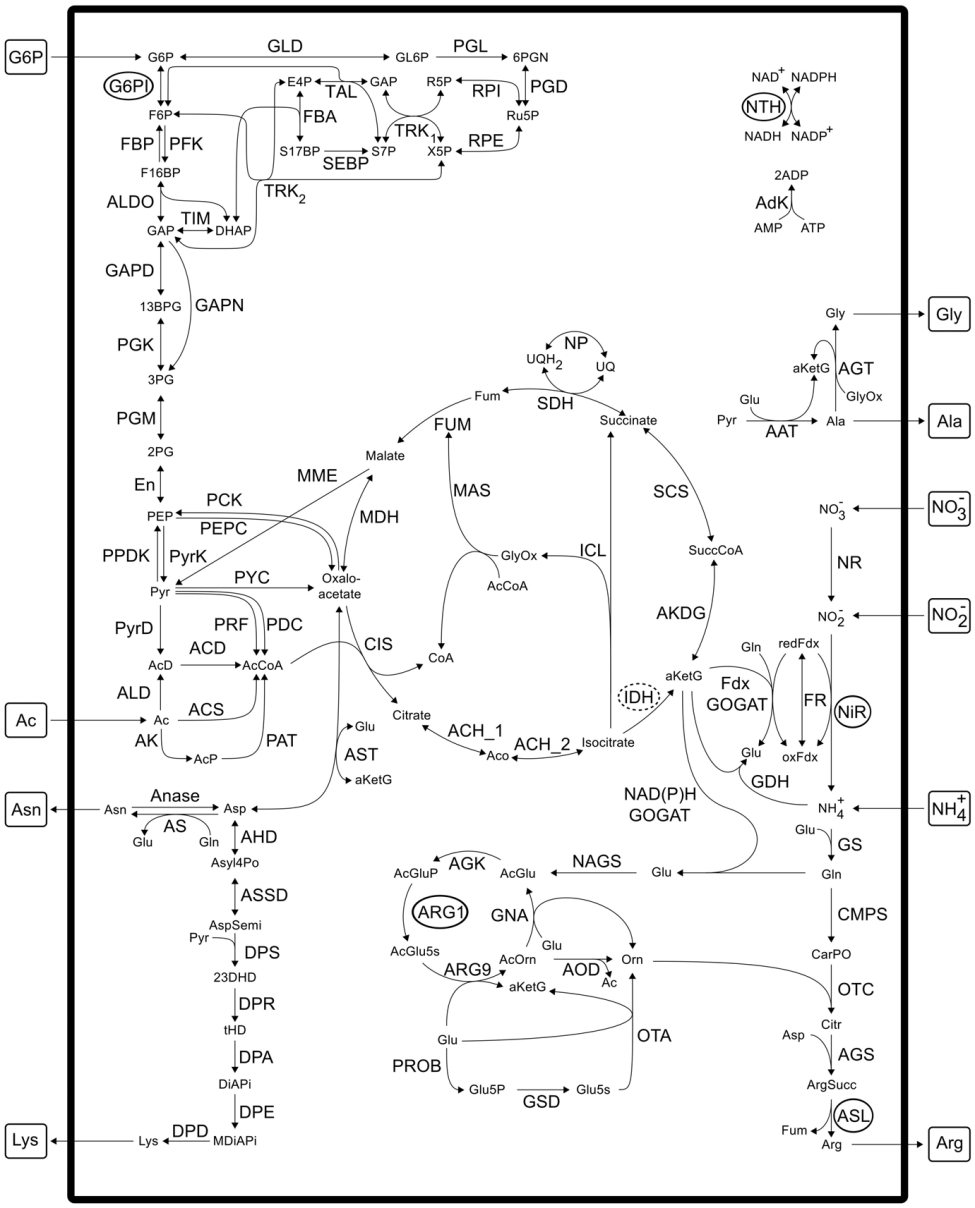
Overview of the reconstructed network of nitrogen metabolism in *C. reinhardtii*. Co-factors such as ATP and NAD(P)H creation or consumption, or CO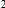, phosphate and water as well as the reactions of pyrophosphatase and the electron transport chain are not shown. For a list of all abbreviations, modelled reactions and species, see Supplementary Tables S1 and S2. External metabolites are framed and enzymes, whose mRNAs are downregulated by CHLAMY1 are encircled. As only the NADPH dependent variant of isocitrate dehydrogenase (IDH) is affected by CHLAMY1, it is marked with a dashed circle.

Taken together, our reconstructed model of nitrogen metabolism of *C. reinhardtii* comprises 105 reactions and 95 metabolites. An overview is given in Fig. 2, while a complete list of reactions can be found in the Supplementary Tables S1 and S2. The sequence analysis revealed that six enzymes are entirely encoded by mRNAs that contain 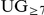-repeats in their 3′ UTRs and are hence presumably under control of CHLAMY1 (Fig. 2).

**Figure 3:**
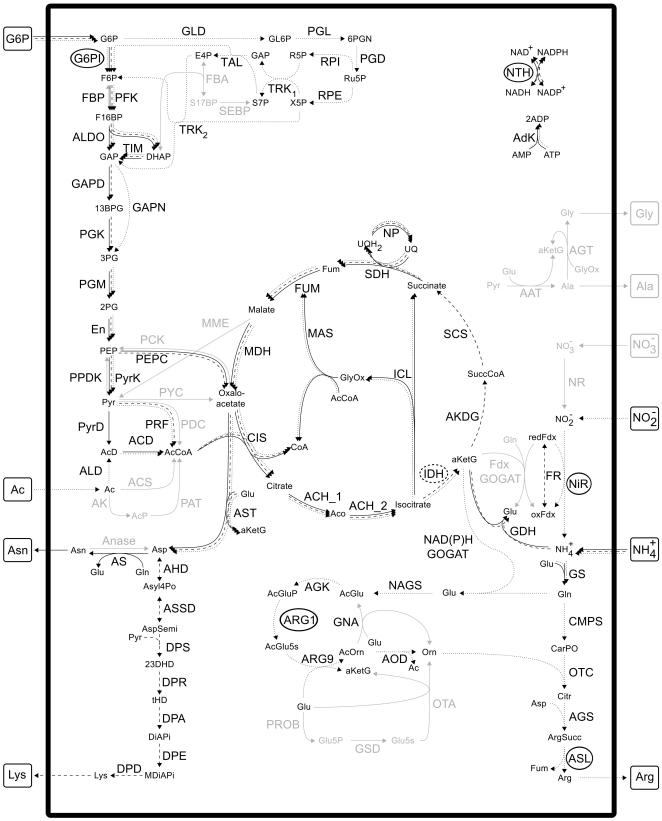
Three example elementary flux modes (EFMs). Solid arrows, most efficient EFM producing asparagine; dashed arrows, most efficient EFM producing lysine; dotted arrows, one selected EFM producing arginine via the pentose phosphate pathway.

The computation of EFMs gave rise to 404252 EFMs for glycine, 684036 EFMs for alanine, 177294 EFMs for asparagine, 406560 EFMs for lysine and 1352352 EFMs for arginine biosynthesis, when G6P as well as acetate were assumed to be available. Three example EFMs are depicted in Fig. 3. The shown EFMs producing asparagine and lysine are the most efficient ones with respect to the yield of amino acids under study per mole carbon source. As for arginine, a less efficient mode is shown to reduce overlap with the other depicted modes and to show another variant, running via the pentose phosphate pathway.

### Maximum carbon yields

To compare the biosynthetic yield of different amino acids, we calculated a so called carbon yield. As described in the Analysis section it was calculated based on the stoichiometric equations of EFMs. It represents the number of carbon atoms in the target amino acid divided by the number of carbon atoms in the carbon source. As beside G6P and acetate, CO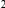 was the only carbon source that was set external, a carbon yield lower than 1 corresponds to a release of CO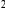 during biosynthesis. In contrast, a carbon yield greater than one corresponds to a non photosynthetic incorporation of CO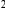.

**Figure 4:**
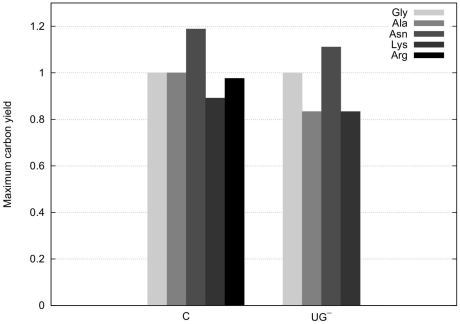
Comparison of maximum carbon yields that are obtained by EFM analysis. The carbon yield was calculated based on stoichiometric equations as described in the Analysis section. It represents the number of carbon atoms in the target amino acid divided by the number of carbon atoms in the carbon source. The comparison is based on ammonium uptake and all possible carbon sources (G6P and acetate). Yields correspond to two different conditions: C, the complete set of enzymes are active at normal rate; 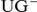, all CHLAMY1 regulated mRNAs and thus, related enzymes are completely inactive. In this all-or-nothing modelling approach, growth on nitrate or nitrite, as well as arginine biosynthesis, is impossible if CHLAMY1 regulation is considered, since nitrite reductase (NiR) and argininosuccinate lyase (ASL) are essential for these processes (see text and Fig. 2).

We compared maximum carbon yields of EFMs for the unperturbed system and the extreme case, where the mRNAs of enzymes under control of CHLAMY1 are completely downregulated. For this analysis, we first computed all EFMs that convert one of the given carbon sources (G6P or acetate) into glycine, alanine, asparagine, lysine or arginine (Fig. 4). During a second run we removed those enzymes whose translation is potentially downregulated during the night by CHLAMY1. As argininosuccinate lyase (ASL) is encoded by an 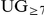-repeat-containing mRNA and subsequently modelled inactive, there are no EFMs for arginine synthesis left under these conditions. Furthermore, since nitrite reductase has the same property, we do no longer find EFMs with nitrate and nitrite consumption if we assume complete downregulation of CHLAMY1 regulated mRNAs and thus, corresponding enzymes. Hence, in this case all EFMs use ammonium as sole nitrogen source.

Beside glycine, the maximum yields for biosynthesis of all amino acids are reduced, if complete downregulation by CHLAMY1 was assumed. However, analysing maximum yields only uses a very small portion of the information about the network's metabolic capabilities. In contrast, EFMs offer a more detailed view on the metabolic capacity. This significant advantage will be exploited below.

### Yield distribution

To make use of the full potential of flux distribution, we first took all EFMs and respective yields into account, rather than analysing solely optimised fluxes with respect to carbon yields. Again, as ASL is a key step in arginine biosynthesis that is inactivated by CHLAMY1, we did not conduct any further analysis of arginine metabolism.

**Figure 5:**
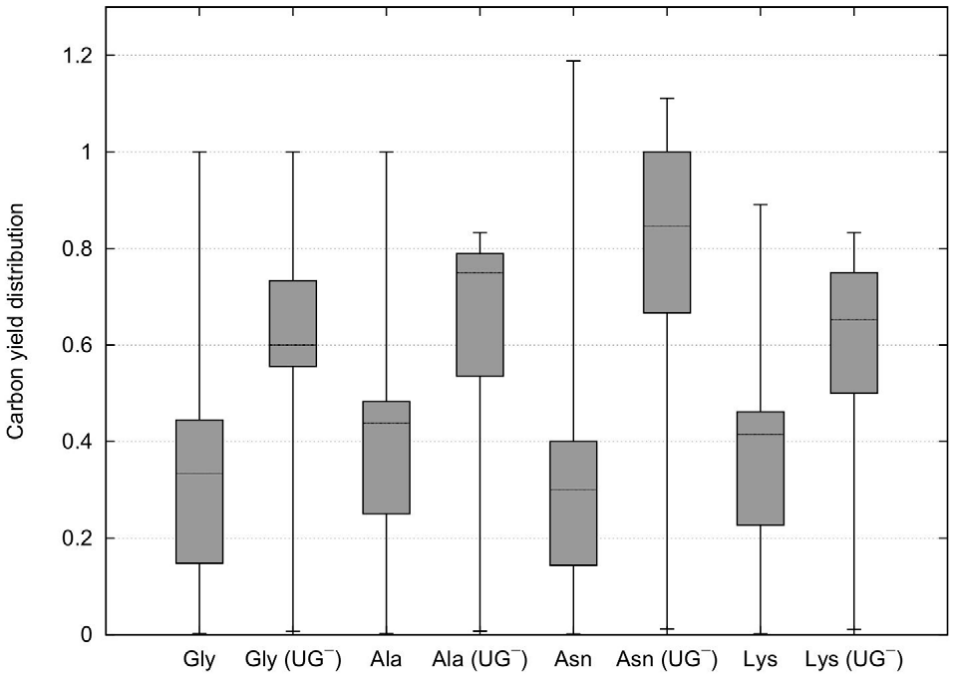
Carbon yield distribution considering complete inactivation. Box plots (with whiskers ranging from minimum to maximum and thick solid line indicating the median) of carbon yield distribution for glycine (Gly), alanine (Ala), asparagine (Asn) and lysine (Lys) associated pathways based on all available carbon sources. Complete inactivation of CHLAMY1 affected reactions is considered here. Knockout of ASL completely inhibits arginine biosynthesis and is hence, not shown (see also Fig. 2). If not marked with 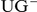, boxplots show complete EFM distribution. Otherwise, they show distribution for all EFMs that are not affected by CHLAMY1 downregulation.

**Figure 6:**
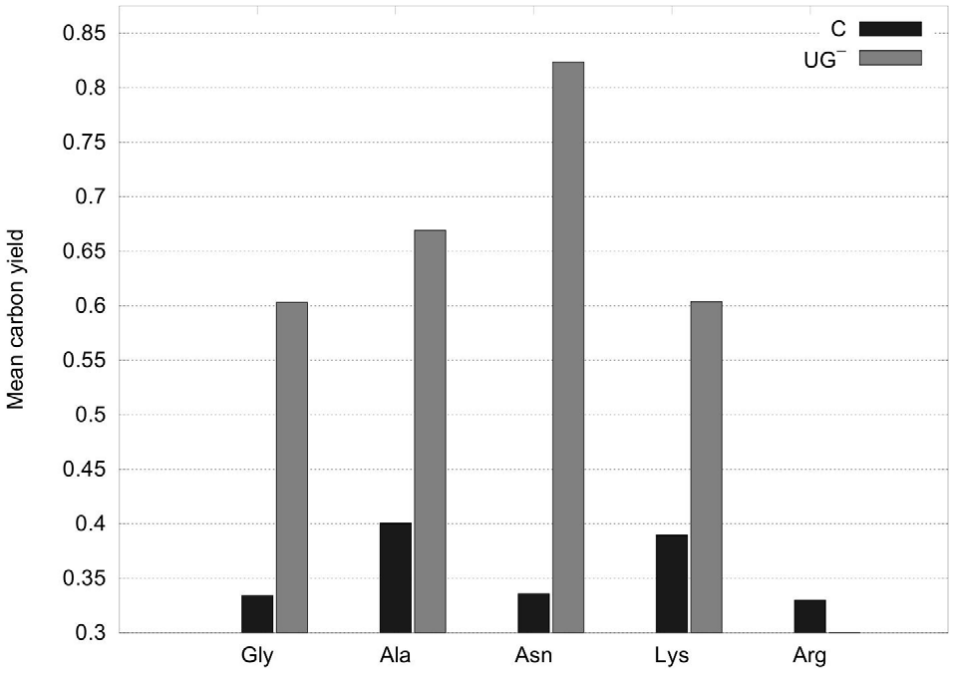
Mean yields assuming complete downregulation by CHLAMY1. In contrast to Fig. 4, the carbon yields of either all EFMs (C) or all those EFMs that do not have CHLAMY1 regulated mRNAs (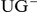) were calculated here. The sum of these yields divided by the number of corresponding EFMs results in the mean yield shown. An increase of the mean yield after downregulation by CHLAMY1 can be observed for all amino acids, except for arginine, as in this case CHLAMY1 downregulates expression of ASL, which is crucial for the arginine pathway (see also Fig. 2).

From Fig. 5 it can be observed that CHLAMY1 predominantly downregulates pathways that have lower yields. Although, the maximum yields decrease the mean carbon yields for all amino acids increase after removing EFMs affected by CHLAMY1 (Fig. 6).

### Weighted influence

Note that until now all EFMs were discarded that contain at least one reaction that is under influence of CHLAMY1. However, such drastic downregulation is questionable and asks for a more realistic modelling.

**Figure 7:**
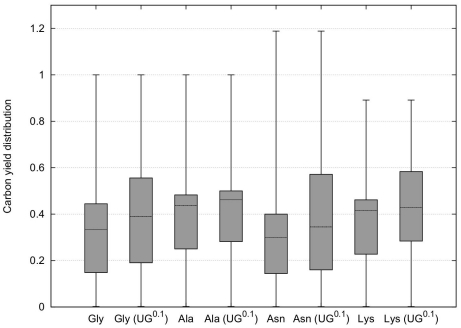
Carbon yield distribution considering partial downregulation. Weighted mean value of carbon yield distribution for the same pathways as in Fig. 5 based on all available carbon and nitrogen sources, considering downregulation of CHLAMY1 affected enzymes to 10%. Either all enzymes are active at normal rate or CHLAMY1 is assumed to downregulate mRNAs with 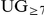-repeat–motif to 10% activity (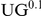) leading subsequently to reduced yield contribution of the affected EFMs.

**Figure 8:**
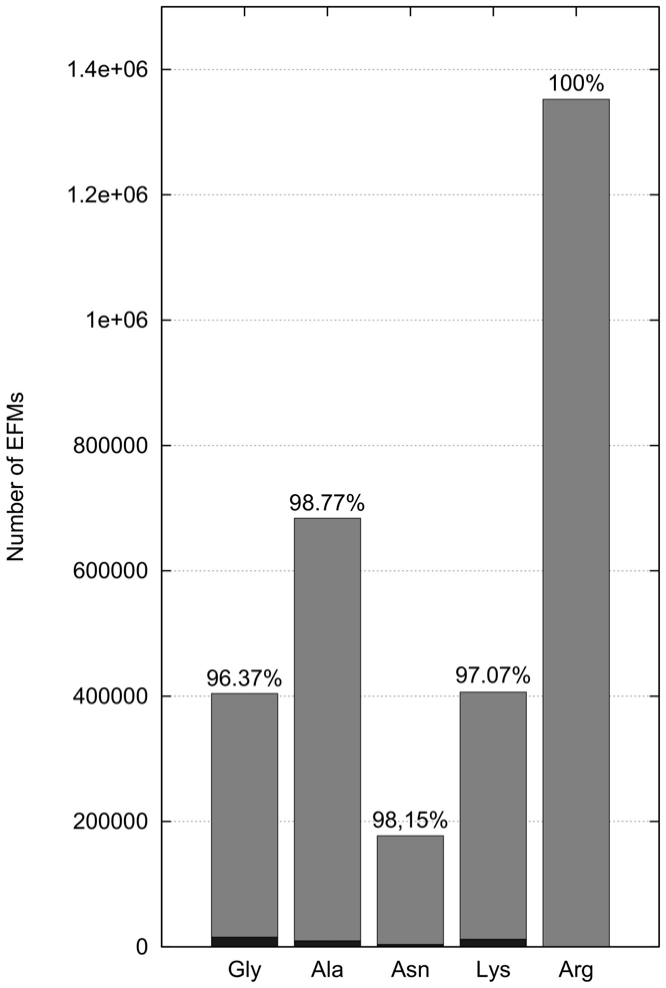
Number of EFMs with and without circadian regulation. More than 96% of all elementary flux modes (gray) are influenced by CHLAMY1. Remaining elementary flux modes, assuming complete downregulation by CHLAMY1, are shown in black.

In the following calculations we therefore circumvented the need to inactivate fluxes, regulated by CHLAMY1, which is usually enforced by EFM analysis. We now assume downregulation of the corresponding fluxes to 10% due to CHLAMY1 binding, rather than complete inactivation. We reduced the impact of inhibited EFMs by weighting EFMs differently, depending on whether they are under CHLAMY1 control or not (Fig. 7). The extent of downregulation by CHLAMY1 is chosen arbitrarily, as corresponding quantitative data is not available. However, reduction factors deviating slightly from 10% do not change the result qualitatively here. Note that downregulation leads to a reduced increase of the interquartile range compared to inactivation (see also Fig. 5). This is due to the large portion of CHLAMY1 controlled fluxes numbering 388832 (96.19%) for glycine, 674436 (98.6%) for alanine, 173543 (97.88%) for asparagine and 394404 (97.01%) for lysine biosynthesis, respectively (Fig. 8). However, this provides a more realistic view on the metabolic state than complete downregulation.

**Figure 9:**
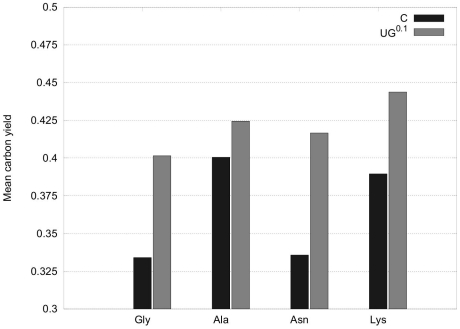
Mean yields assuming partial downregulation of genes by CHLAMY1. All EFMs have been used to calculate a weighted mean according to Eq. 3. An increase of the weighted mean yield can again be observed for all amino acids as in Fig. 6. However, the increase is less pronounced.

Additionally, we calculated the mean of molar yields by using the formula for weighted means given by Eq. (3). This enables us to weight every derived yield and hence, also the underlying flux, represented by its respective EFM. We applied a weight of 10% to EFMs affected by CHLAMY1 and a unity weight to all remaining fluxes. The resulting mean yield is considerably increased upon CHLAMY1 binding (Fig. 9).

## Discussion

In this study, we have outlined a method that interconnects sequence based knowledge with metabolic pathway analysis. The method has been illustrated by nitrogen metabolism of *C. reinhardtii*, which is under the control of the circadian clock. The calculated elementary flux modes provide a data set that is well-suited for quantifying and understanding the complex architecture of this network. The large number of modes (e. g. 1352352 for arginine) point to a considerable redundancy of this network. Intriguingly, our results show that downregulation of circadian controlled enzymes improves carbon distribution and thus, decreases energy consumption. The somewhat counter-intuitive result that knocking out or downregulating several enzymes may lead to an increase in average yield arises, because poor pathways are deleted or downregulated, so that more efficient pathways become more dominant. A similar phenomenon was observed earlier in the context of strain optimisation [9], [27], [28]. Our approach focuses on analysing altered fluxes due to regulatory influences in general. We compare two or more physiological situations (e. g. day- and night-time) rather than manipulated setups. Other examples may be provided by hibernation vs. summertime stage or different developmental stages such as embryonic vs. adult. The regulatory information can be provided in a wide variety of forms, including transcriptional regulatory events as time-dependent constraints [29].

Additionally, we have considered information derived from sequence data. Since it is known that many regulatory proteins bind to specific motifs in the mRNA or to promoters, such information is extremely useful in modelling regulation of metabolism. Moreover, measuring fluxes in detail is already a demanding task for simple model organisms, like *Escherichia coli* or *Saccharomyces cerevisiae*, but might be virtually infeasible for higher organisms, when regulatory complexity becomes more sophisticated. Thus, our approach proves to be an easy-to-use, helpful method to determine the type and impact of influences of regulatory factors.

**Figure 10:**
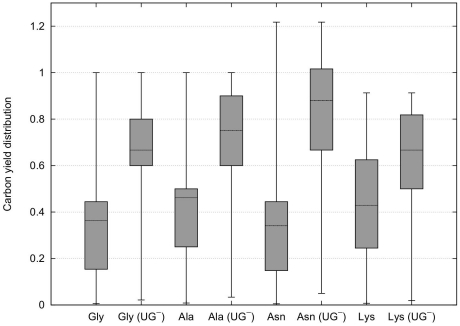
Yield distribution for GAP and acetate as carbon source. Carbon yield distribution was calculated assuming complete inactivation. In contrast to Fig. 5 GAP and acetate were used as carbon source and the maximum yields (upper whiskers) do not change between EFM sets including all enzyme or those that are not affected by CHLAMY1 downregulation (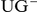). Box plots (with whiskers ranging from minimum to maximum and thick solid line indicating the median) of carbon yield distribution for glycine (Gly), alanine (Ala), asparagine (Asn) and lysine (Lys) associated pathways based on all available carbon sources.

Considering only the maximum carbon yields of pathways summarised in Fig. 4, indicates that *C. reinhardtii* remains able to synthesise glycine, alanine, asparagine and lysine but with reduced theoretical effectiveness while not being able to synthesis arginine if one assumes complete downregulation by CHLAMY1 at night-time. However, as there are more than three million possible routes within the network producing the target amino acids and the main portion of all EFMs (above 96% for all amino acids, see also Fig. 8) is affected by CHLAMY1 action, solely focusing on maximum carbon yields provides a limited view and would lead to misinterpretations. Furthermore, the calculation of the maximal yield is sensitive to the size of the model and the carbon sources chosen. If we use glyceraldehyde-3-phosphate (GAP) and acetate as carbon source and thus, remove glycolysis and the pentose phosphate pathway from the model, the maximum yield does not change between sets of EFMs with and without CHLAMY1 affected reactions (see Fig. 10).

To study the spectrum of metabolic capabilities, we analysed the whole yield distribution. The results, shown in Fig. 5, reveal that CHLAMY1 influences the mRNA expression of enzymes mainly taking part in EFMs that realise low yields. Thus, translational downregulation by CHLAMY1 during the night leads to an increased median yield for the considered amino acid production whereas the maximum yield decreases.

During night-time, photosynthesis is impossible and, hence, energy is largely limited. A prohibition of energy-consuming reactions that usually contribute to low carbon yields during the night has already been observed experimentally for *Arabidopsis thaliana* [30]. The decrease in maximum carbon yield observed in our analysis is mainly due to the fact that G6PI is regulated by CHLAMY1 and thus, G6P is forced to enter the pentose phosphate pathway (PPP). This might be necessary as the PPP is required for the synthesis of nucleotides. As DNA-replication occurs preferentially during the night, this regulatory compromise can be considered as an optimised outcome of evolution.

Taken together, our results are in good agreement with experimental observations and evolutionary considerations. In contrast to the dependency of the decrease of the maximum yields on the model size and carbon source chosen, the increase of the yield distribution can be found for both G6P and GAP as carbon source (see Fig. 5 and Fig. 10, respectively).

**Figure 11:**
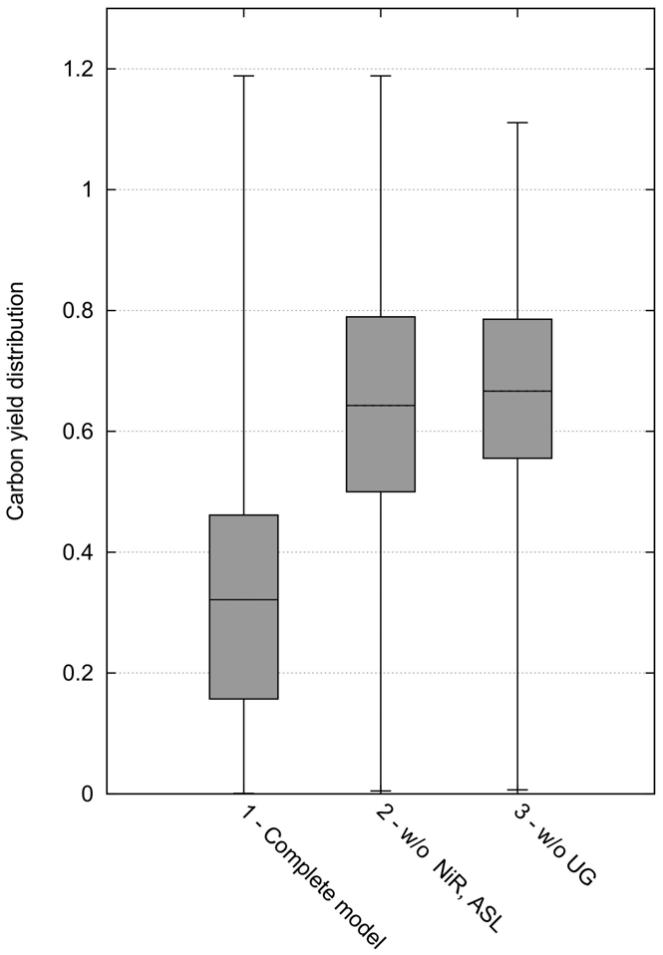
Combined carbon yield distribution for complete downregulation by CHLAMY1. For the depicted boxplots the yield distribution for all amino acids including arginine have been combined into one yield distribution. Box plots (with whiskers ranging from minimum to maximum and thick solid line indicating the median) of carbon yield distribution are based on all available carbon and nitrogen sources. Complete inactivation of CHLAMY1 affected enzymes is considered here.

Beside ASL and NiR, CHLAMY1 regulated enzymes are identified based on UG-repeats found in the annotated 3′ UTR of the respective genes. For ASL and NiR CHLAMY1 binding has been shown experimentally and the introduction of the respective 3′ UTR sequences into luciferase constructs lead to a robust circadian enzyme activity [25]. Furthermore, NiR activity has been shown to cycle in circadian manner [26]. To analyse how the prediction of CHLAMY1 regulated enzymes based on sequence analysis might influence our results we calculated the yield distribution with only ASL and NiR downregulated by CHLAMY1 for comparison. As Fig. 11 shows, NiR and ASL are the enzymes mainly contributing to an increased yield. As G6PI is still active in this case the maximum yield is equal to that of the complete model. This again demonstrates that the calculation of the maximum yield alone is relatively sensitive to changes in the model and may lead to misinterpretations.

The analysis presented here was restricted to the influence of CHLAMY1 regulation on metabolism. There might be other processes influencing the circadian regulation of nitrogen metabolism like transcriptional and posttranslational regulation. It has been described that the transcription of some enzymes included in our model are regulated in a circadian manner [31]. However, all enzymes described in the aforementioned approach have isoenzymes that are not under the control of the circadian clock. As we did not distinguish between different isoforms as long as they use the same cofactors, an inclusion of transcriptional regulation would not affect our simulations. Furthermore, we did not include any compartmentalisation in our model, as due to the resulting complexity the calculation of all EFMs would not be feasible.

Here, we have assumed that all fluxes contribute with equal probability to an overall flux, as done earlier in the case of incomplete knowledge [11]. This assumption probably does not describe reality properly. However, it allows one to analyse the robustness and full flexibility against altered environmental conditions. Moreover, it enables us to predict qualitative changes of the metabolic system under investigation. Furthermore, it has been noticed that approaches based on optimality principles are dependent on the applied constraints [13], [32]–[34] and matching them to experimental results meets with various difficulties [35], [36]. As we have shown in this study, weighting EFMs affected by regulating factors differently from unaffected EFMs, preserves the EFM inherent yield, while changing the overall yield distribution (Fig. 7). Additionally, computing a weighted arithmetic mean of all carbon yields provides valuable information about effects of the regulating factors, while circumventing artificial all-or-none simulations. The simplicity of this approach provides the advantage that no parameters, like reaction rates, are required and no additional assumption have to be made.

Further analysis of the calculated EFMs shows that only approximately 1/50th of the original set of EFMs is still fully active after CHLAMY1 binding. Therefore, the metabolic flux through the system is considerably reduced during the night-time, which is in line with the reduction of carbon and energy consumption when photosynthesis is inactive. This holds independently of the carbon source chosen. Particularly EFMs with a low yield are suppressed, so that the average yield increases. If CHLAMY1 binding is reduced at the end of the night resulting in the expression of target enzymes at the beginning of the day when photosynthetic energy is again available, the metabolic capability and robustness of nitrogen metabolism is greatly increased and allows fast incorporation of nitrogen into the organism. As energy is no longer limiting, there is no need to restrict to those reactions with high yields and low energy consumption. Therefore, CHLAMY1 binding during the night appears to ensure energy conservation while still allowing nitrogen fixation. Due to the stabilisation of mRNA by CHLAMY1 and release at the end of the night [23], [25], [26], it furthermore enables a high metabolic capacity as soon as enough energy is available.

Fig. 7 reveals that downregulation rather than inactivation of CHLAMY1 affected reactions, still leads to an increase in global carbon yields, although the increase is remarkably lower. This is due to the large portion of CHLAMY1 influenced fluxes.

In general, using weighted influences instead of the simplified all-or-none approach, can be used to study the impact of two regulators leading to different residual activity of enzymes. Furthermore, it could also be used to interpret microarray or other expression data. Here, fold changes could be used as weighting factors to simulate metabolic changes of a given system. Hence, it provides a useful tool to connect the growing amount of high throughput expression data to pathway analysis.

## Analysis

### Calculating amino acid composition

The amino acid compositions of selected organisms were derived from complete genome open reading frame (ORF) prediction data in fasta file format. The fasta files from *Homo sapiens*, *Mus musculus*, and *Arabidopsis thaliana* were obtained from the UniProt database [37], while the fasta file for *Chlamydomonas reinhardtii* was fetched from a database provided by the Joint Genome Institute [38]. These files were scanned for total amino acid distribution and the results summarised in Fig. 1.

### Pathway reconstruction

A metabolic network comprising the nitrogen metabolism in *C. reinhardtii* (Fig. 2) was reconstructed using the KEGG [39] and ChlamyCyc [40] databases, the biochemical pathways textbook [41] as well as bibliomic data. All reactions were manually curated, which included mass balancing if required and verification of reaction reversibility based on existing biochemical knowledge. If the irreversibility was not conclusive, we set the corresponding reaction reversible.

The carbohydrate metabolism under study includes glycolysis, gluconeogenesis, the pentose phosphate pathway, acetate uptake, the citrate cycle and the glyoxylate shunt. The nitrogen uptake model was reconstructed using data from [42]–[45]. Moreover, the biosynthetic pathways of glycine, alanine, asparagine, lysine and arginine, which provide the target metabolites of the model, are taken into account by comparing charts [41] with the above-mentioned databases and biological literature. The accessible carbon sources are acetate, simulating heterotrophic growth, and glucose-6-phosphate (G6P), resulting from starch breakdown during the night. Moreover, molecular nitrogen is provided by nitrate, nitrite or ammonium uptake. Consequently, those substances as well as G6P, acetate and the five above-mentioned amino acids are modelled as external metabolites, that is, their concentrations are considered to be buffered. In contrast, we modelled all energy and redox carriers, such as ATP, NAD(P)H and ferredoxin, as internal. The network in SMBL A SBML version of the network is provided in the Supplements.

### Sequence analysis

The mRNA sequences that are associated to the enzymes included in the model were analysed for perfect 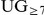-repeats (UG UG UG) in annotated 3′ UTR [22] of models from the Joint Genome Institute database version 4.0 of *C. reinhardtii* [38].

Special emphasis had to be put on isoenzymes, as in several cases mRNAs encoding enzymes contained 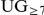-repeats, while others associated to enzymes catalysing the same reaction did not. As we did not regard localisation of enzymes and did not distinguish between isoenzymes as long as they use the same cofactors, the corresponding reactions were simulated not to be under control of the circadian clock via CHLAMY1 for the EFM analysis.

### Computation of elementary flux modes

EFMs were computed with efmtool [46] inside the MATLAB environment, version 2008b (The MathWorks, Natick, MA, USA). Details of elementary flux mode calculation are described elsewhere [46], [47].

### Calculation of yields

In order to compare the effectiveness of different modelled amino acid pathways, we computed carbon yields according to:
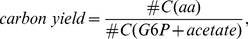
(1)where 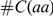 and 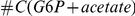 refer to the numbers of carbon atoms in the considered target amino acid (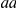) and in the substrates 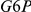 and 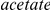, respectively. The number of carbon atoms were obtained from the overall chemical equation of each elementary mode. The mean yields 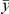 of all yields 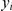 were calculated according to standard formula for mean calculation:
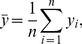
(2)


### Weighted yields

To calculate the effect of CHLAMY1 downregulation rather than full inactivation of the influenced enzymes, yields from EFMs were weighted differently in the resulting yield distribution. We arbitrarily assumed downregulation to 10% as experimental measures for the degree of downregulation are not available. For the calculation of the yield distribution and visualisation in boxplot graphics the yields unaffected by CHLAMY1 were counted ten times, whereas yields affected by that regulator were only taken into account once.

Additionally, we calculated weighted mean yields for a simplified visualisation of the downregulating effect of CHLAMY1. To do so we computed the weighted arithmetic mean yield 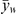 according to the following equation:
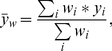
(3)with 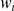 being the weight of the derived carbon yield 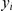. These weighting factors can be defined in various ways. Here, we use the definition given above based on the fractional extent of downregulation.

## Supporting Information

Table S1 Overview of modelled metabolites and corresponding abbreviations.
(PDF)

Table S2Overview of modelled enzymes and corresponding EC–numbers, abbreviations as well as JGI database IDs (cre v4.0; http://genome.jgi-psf.org/Chlre4/). The code of the UG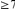-repeats is as follows: i – intron, e – exon, 5′/3′ UTR – the 5′ or 3′ untranslated region of an enzyme. For bold marked UG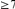-repeat entries CHLAMY1 binding has been shown experimentally.(PDF)
