# Supplementary material for: Predicting the Physiological Role of Circadian Metabolic Regulation in the Green Alga Chlamydomonas reinhardtii
Source: PLoS One. 2011 Aug 22;6(8):e23026. doi: 10.1371/journal.pone.0023026 (PMC3161734; doi:10.1371/journal.pone.0023026)
Supplement: Table S1 — Overview of modelled metabolites and corresponding abbreviations. (PDF) [file pone.0023026.s001.pdf]

Supplementary Material

Table S1

Abbreviation	Name
13BPG	1,3-Bisphosphoglycerate
23DHD	2,3-dihydrodipicolinate
2PG	2-Phospho-glycerate
3PG	3-Phospho-glycerate
6PGN	6-Phospho-gluconate
Ac	Acetate
AcCoA	Acetyl-coenzyme-A
AcD	Acetaldehyde
AcGlu	Acetyl-glutamate
AcGlu5s	Acetyl-glutamate 5-semialdehyde
AcGluP	Acetylglutamyl-phosphate
Aconitate	Aconitate
AcOrn	Acetyl-ornithine
AcP	Acetylphosphate
ADP	Adenosine diphosphate
aKetG	α-Ketoglutarate
Ala	Alanine
AMP	Adenosine monophosphate
Arg	Arginine
ArgSucc	Arginino-succinate
Asn	Asparagine
Asp	Aspartate
AspSemi	Aspartate-semialdehyde
Asyl4Po	Aspartyl-4-phosphate
ATP	Adenosine triphosphate
$biPO_4^{3-}$	Pyrophosphate
CarPO	Carbamoyl-phosphate
Citr	Citrulline
Citrate	Citrate
CO_2	Carbon dioxide
CoA	Coenzyme A
DHAP	Dihydroxyacetone-phosphate

continued on next page

Abbreviation	Name
DiAPi	L,L-Diaminopimelate
E4P	Erythrose-4-phosphate
F16BP	Fructose-1,6-biphosphate
F6P	Fructose-6-phosphate
Fum	Fumerate
G6P	Glucose-6-phosphate
GAP	Glyceraldehyde-3-phosphate
GL6P	Glucono-1,5-lactone-6-phosphate
Gln	Glutamine
Glu	Glutamate
Glu5P	Glutamate-5-phosphate
Glu5s	Acetyl-glutamate-5-semialdehyde
Gly	Glycine
GlyOx	Glyoxylate
H	Hydrogen
H_2O	Water
HCO_3^-	Hydrogencarbonate
Isocitrate	Isocitrate
Lys	Lysine
Malate	Malate
MDiAPi	Meso-diaminopimelate
NAD(H)	Nicotinamide adenine dinucleotide
NADP(H)	Nicotinamide adenine dinucleotide phosphate
NH_4^+	Ammonium
NO_2^-	Nitrite
NO_3^-	Nitrate
Orn	Ornithine
Oxaloacetate	Oxaloacetate
oxFdx	oxidized Ferredoxin
PEP	Phosphoenolpyruvate
PO_4^{3-}	Phosphate
Pyr	Pyruvate
R5P	Ribose-5-phosphate
redFdx	reduced Ferredoxin
Ru5P	Ribulose-5-phosphate
S17BP	Sedoheptulose-1,7-bisphosphate

continued on next page

**Abbreviation**	**Name**
S7P	Sedoheptulose-7-phosphate
SuccCoA	Succinylcoenzyme-A
Succinate	Succinate
tHD	Tetrahydrodipicolinate
UQ	Ubiquinone
UQH_2	Ubiquinol
X5P	Xylulose-5-phosphate
