# Supplementary material for: Predicting the Physiological Role of Circadian Metabolic Regulation in the Green Alga Chlamydomonas reinhardtii
Source: PLoS One. 2011 Aug 22;6(8):e23026. doi: 10.1371/journal.pone.0023026 (PMC3161734; doi:10.1371/journal.pone.0023026)
Supplement: Table S2 — Overview of modelled enzymes and corresponding EC–numbers, abbreviations as well as JGI database IDs (cre v4.0; http://genome.jgi-psf.org/Chlre4/). The code of the UG-repeats is as follows: i – intron, e – exon, 5′/3′ UTR – the 5′ or 3′ untranslated region of an enzyme. For bold marked UG-repeat entries CHLAMY1 binding has been shown experimentally. (PDF) [file pone.0023026.s002.pdf]

Supplementary Material

Table S2

**Abbreviation**	**Name**	**EC-number**	**JGI–ID**	**UG$_{\geq 7}$-repeat**
Anase	Asparaginase	3.5.1.1	187983	3*i
ACD	Acetaldehyde Dehydrogenase	1.2.1.10	133318	1*i
AdK	Adenylate Kinase	2.7.4.3	24512	
			103322	
			114363	
			118113	1*i
			129362	
			133184	
			194134	
			194947	
			196741	
ACH	Aconitate Hydratase	4.2.1.3	129025	
			195293	
ACS	Acetyl CoA Synthetase	6.2.1.1	196311	1*i
			119897	1*i
			391501	
			194063	
AGK	Acetylglutamate Kinase	2.7.2.8	78991	
			143603	1*i
AGS	Argininosuccinate Synthase	6.3.4.5	58140	
AGT	Alanine-glyoxylate transaminase	2.6.1.44	133057	
			194541	
			205967	1*i
AHD	Aspartate Kinase	2.7.2.4	161455	
			196316	
			196925	
AK	Acetate Kinase	2.7.2.1	128476	1*i
			129982	1*i
AKDG	α-Ketoglutarate Dehydrogenase Complex	1.8.1.4	57890	1*i
		1.8.1.5	205763	
		2.3.1.61	145395	
		2.3.1.62	145987	

continued on next page

Abbreviation	Name	EC-number	JGI–ID	$UG_{\geq 7}$-repeat
AKDG	α-Ketoglutarate Dehydrogenase Complex	1.2.4.2	79471	
ALD	Aldehyde Dehydrogenase	1.2.1.3	135609	1*i
ALDO	Aldolase	4.1.2.13	24459	
			29185	
			152892	
			196304	
AOD	Acetylornithinase	3.5.1.16	155546	
ARG1	Acetyl-gamma-glutamyl-phosphate Reductase	1.2.1.38	191987	1*i, 1*3' UTR
ARG9	Acetylornithine Aminotransferase	2.6.1.11	119395	
			139007	
AS	Asparagine Synthase	6.3.5.4	140252	
			167865	
ASL	Argininosuccinate Lyase	4.3.2.1	101662	**1*3' UTR**
ASSD	Aspartate Semialdehyde Dehydrogenase	1.2.1.11	148810	
AST	Aspartate Aminotransferase	2.6.1.1	118364	2*i
			126943	2*i
			129557	
			146923	
			170056	1*i
			174097	2*i
			186959	2*i
			206390	2*i
BP	Inorganic Pyrophosphatase	3.6.1.1	133620	
			137778	
			174103	
CIS	Citrate Synthase	2.3.3.1	24263	2*i
			194915	
CMPS	Carbamoyl-phosphate Synthase	6.3.5.5	128227	
			195255	
DPA	Diaminopimelic Acid Aminotransferase	2.6.1.-	129557	
DPD	Diaminopimelate Decarboxylase	4.1.1.20	146886	3*i
DPE	Diaminopimelate Epimerase	5.1.1.7	150957	
DPR	Dihydrodipicolinate Reductase	1.3.1.26	205760	
DPR	Dihydrodipicolinate Reductase	1.3.1.26	395433	1*i
DPS	Dihydrodipicolinate Synthase	4.2.1.52	126518	

continued on next page

Abbreviation	Name	EC-number	JGI–ID	UG$_{\geq 7}$-repeat
En	Enolase	4.2.1.11	83064	
FBA	Sedoheptulose-Bisphosphate Aldolase	4.1.2.-	24459	
FBP	Fructose-1,6-Bisphosphatase	3.1.3.11	24084	
Fdx GOGAT	Glutamate Synthase, Ferredoxin-dependent	1.4.7.1	140487	
FR	Ferredoxin Reductase		195553	
FUM	Fumarate Hydratase	4.2.1.2	195953<br>282848	
G6PI	Phosphoglucose Isomerase	5.3.1.9	135220	1*3' UTR
GAPD	Glyceraldehyde-3-phosphate Dehydrogenase	1.2.1.12	16652<br>102889<br>129019<br>140618<br>153894<br>195910<br>196442<br>196443	2*i
GAPN	Triosephosphate Dehydrogenase	1.2.1.9	102889	
GDH	Glutamate Dehydrogenase	1.4.1.3	82916<br>137469	2*i<br>1*i
GLD	Glucose-6-phosphate-1-Dehydrogenase	1.1.1.49	119861<br>309903<br>173841	1*i, 1*e
GNA	Glutamate Acetyltransferase	2.3.1.35	288806	3*i
GS	Glutamine Synthetase	6.3.1.2	129468<br>133971<br>136895<br>147468	1*3' UTR<br><br><br>1*3' UTR
GSD	Glutamate-5-semialdehyde Dehydrogenase	1.2.1.41	130812	
ICL	Isocitrate Lyase	4.1.3.1	104431<br>191668	1*i
IDH	Isocitrate Dehydrogenase ($NADP^+$-dependent)	1.1.1.42	196567	1*3' UTR
	Isocitrate Dehydrogenase (NAD^+-dependent)		196042	

continued on next page

Abbreviation	Name	EC-number	JGI–ID	$UG_{\geq 7}$-repeat
IDH	Isocitrate Dehydrogenase (NAD^+-dependent)		196044	1*i
MAS	Malate Synthase	2.3.3.9	196328	1*i
MDH	Malate Dehydrogenase	1.1.1.37	60444	
			126023	
			137163	1*i
			158129	
MME	Malate Dehydrogenase ($NADP^+$ dep.)	1.1.1.40	126820	
			147722	3*i
			196351	3*i
			196831	2*i
			196832	
MME	Malate Dehydrogenase (NAD^+ dep.)	1.1.1.38	196833	
NAD(P)H GOGAT	Glutamate Synthase, NAD(P)H-dependent	1.4.1.14	205746	
NAGS	Acetylglutamate Synthase	2.3.1.1	130199	
			288806	2*i
NiR	Nitrite Reductase	1.7.7.1	192085	6*i, 3' UTR
NK	NAD^+ Kinase	2.7.1.23	123446	1*i
			165793	
			196779	
NP	NADH-ubiquinone oxidoreductase	1.6.5.3	24195	
			54440	
			57090	
			58686	
			59411	
			77311	
			79362	
			127317	
			127639	
			131464	
			132151	
			132909	
			135635	
			139850	
			143441	

continued on next page

Abbreviation	Name	EC-number	JGI–ID	$UG_{\geq 7}$-repeat
NP	NADH-ubiquinone oxidoreductase	1.6.5.3	145512	
			149240	
			164272	
			164424	
			174569	2*i
			182226	
			182302	
			182980	
			184222	
			184606	1*i
			185013	
			186185	
			186342	
			187994	
			188142	
			190543	
			190916	
			191146	
			193762	
			194458	
			195711	1*i
NR	Nitrate Reductase	1.7.1.1	184661	2*i
NTH	$NAD(P)^+$ Transhydrogenase	1.6.1.2	139758	1*3' UTR
OTA	Ornithine Transaminase	2.6.1.13	195386	
OTC	Ornithine Transcarbamylase	2.1.3.3	188762	
PAT	Phosphate Acetyltransferase	2.3.1.8	11226	3*i
			191051	
PCK	Phosphoenolpyruvate Carboxykinase	4.1.1.49	196612	1*i
PDC	Pyruvate Dehydrogenase Complex	1.8.1.4	57890	1*i
			205763	
		2.3.1.12	145395	
			149206	
			149709	
			187285	
			196500	

continued on next page

Abbreviation	Name	EC-number	JGI–ID	$UG_{\geq 7}$-repeat
PDC	Pyruvate Dehydrogenase Complex	1.2.4.1	139515	
			193810	1*i
			196469	
			206010	1*e/3' UTR
			190446	1*i
			155587	1*i
PEPC	Phosphoenolpyruvate Carboxylase	4.1.1.31	80312	2*i
			182821	
PFK	Phosphofructokinase	2.7.1.11	411593	2*i
			196310	
			196430	1*i
			196624	
PGD	6-Phosphogluconate Dehydrogenase	1.1.1.44	115511	
			120516	
			128576	
			158911	
			192597	
PGK	Phosphoglycerate Kinase	2.7.2.3	36313	
			132210	
			196383	
PGL	6-Phosphogluconolactonase	3.1.1.31	146607	
			390565	1*i
PGM	Phosphoglycerate Mutase	5.4.2.1	8761	
			21373	2*i
			30383	
			325517	
			119977	
			161085	
			196305	
PPDK	Pyruvate-phosphate Dikinase	2.7.9.1	206694	
			196616	
PRF	Pyruvate-Ferredoxin Oxidoreductase	1.2.7.1	206677	
PROB	Glutamate-5-kinase	2.7.2.11	170370	
			189050	
PYC	Pyruvate carboxylase	6.4.1.1	402089	1*i
PyrD	Pyruvate Decarboxylase	4.1.1.1	127786	1*i
PyrK	Pyruvate Kinase	2.7.1.40	104490	

continued on next page

Abbreviation	Name	EC-number	JGI–ID	$UG_{\geq 7}$-repeat
PyrK	Pyruvate Kinase	2.7.1.40	107530	
			118203	
			119280	
			122254	
			136854	
			149896	
			196263	
			196261	
			196263	
RPE	Ribulose-5-phosphate 3-Epimerase	5.1.3.1	6964	
			135614	
RPI	Ribose-5-phosphate Isomerase	5.3.1.6	55838	
			205912	
SCS	Succinate CoA Synthetase	6.2.1.5	24101	1*i
			56839	
			196569	1*i
			196570	
SDH	Succinate Dehydrogenase	1.3.5.1	142231	1*i
			183570	
			195641	
			394775	2*i
TAL	Transaldolase	2.2.1.2	146574	
			176076	4*i
			287436	
TIM	Triosephosphate Isomerase	5.3.1.1	26265	
TRK_1 / TRK_2	Transketolase	2.2.1.1	141319	
